# Catalyst System for Hydrogenation Catalysis Based on Multiarm Hyperbranched Polymer Templated Metal (Au, Pt, Pd, Cu) Nanoparticles

**DOI:** 10.3390/polym9090459

**Published:** 2017-09-19

**Authors:** Yunfeng Shi, Lixin Liu, Fengyue Zhang, Mengyuan Niu, Yanzhu Zhao, Yifan Fan, Yanping Liang, Mei Liu, Zhenzhu Zhang, Junjie Wang

**Affiliations:** 1 School of Chemistry and Chemical Engineering, Anyang Normal University, Anyang 455000, China; slizn2002@163.com (L.L.); 18738291636@163.com (F.Z.); 15083142320@163.com (M.N.); zyzsgxn@163.com (Y.Z.); 18625768213@163.com (Y.F.); 18749327281@163.com (Y.L.); 17839164457@163.com (M.L.); 18738292880@163.com (Z.Z.); 2 Henan Province Key Laboratory of New Optoelectronic Functional Materials, Anyang Normal University, Anyang 455000, China

**Keywords:** multiarm hyperbranched polymer, metal nanoparticles, hydrogenation catalysis

## Abstract

With a hyperbranched poly(amidoamine) core and many water-soluble poly(ethylene glycol) monomethyl ether arms connected by pH-sensitive acylhydrazone bonds, multiarm hyperbranched polymer was used as nanoreactor and reductant to prepare metal nanoparticles endowed with intelligence and biocompatibility. The multiarm hyperbranched polymer encapsulated nanoparticles (NPs) showed excellent catalytic activity for hydrogenation, thus an excellent catalyst system for hydrogenation was established. The rate constants could reach as high as 3.48 L·s^−1^·m^−2^, which can be attributed to the lack of surface passivation afforded by the multiarm hyperbranched polymer.

## 1. Introduction

Hyperbranched polymers are a kind of highly branched polymers with three-dimensional topological structures. They have many inner cavities and a large amount of terminal functional groups. Due to their unique molecular structures and facile synthesis route, hyperbranched polymers have received much attention and remarkable development has been made on their synthesis, characterization, and modification in the past decades [1,2,3,4]. This leads to an increasing interest on their application, such as molecular self-assembly [5,6,7,8,9,10,11,12], control-release of dyes [13,14,15,16,17,18], drug delivery [19,20,21], protein delivery [22,23], gene transfection [24,25,26,27,28], molecular imaging [29], nanoparticle (NP) encapsulation [30,31,32,33], and so on.

NP synthesis is one of the most important applications of hyperbranched polymers in recent years. With a three-dimensional globular architecture, numerous cavities, and plenty of peripheral functional groups [34,35,36], hyperbranched polymers are regarded as ideal stabilizers and nanoreactors/templates to prepare size-controlled semiconductors [37,38,39,40,41], metal nanoparticles (NPs) [42,43,44,45,46,47], metal nanodots [48,49], and metal oxide nanocrystals [25,26,50,51], etc. Until now, various hyperbranched polymers, not only the conventional covalent hyperbranched polymers but also supramolecular multiarm hyperbranched polymers [41,52] have been reported to prepare nanocrystals. By preparing nanocrystals within hyperbranched polymers, the environmental sensitivity and biocompatibility of polymers [53,54], and the optical, magnetic, and electrical properties of nanocrystals can be combined, endowing nanocrystals with intelligence and biocompatibility.

In this work, multiarm hyperbranched polymers were used as reductants, stabilizers, and nanoreactors to prepare metal (Au, Pt, Pd, Cu) NPs, resulting in an excellent catalyst system for hydrogenation catalysis. The multiarm hyperbranched polymer (HPAMAM-g-MPEG) has a hyperbranched poly(amido amine) (HPAMAM) core and many water-soluble poly(ethylene glycol) monomethyl ether (MPEG) arms connected by pH-sensitive acylhydrazone bonds. The multiarm hyperbranched polymers with acylhydrazone bonds have a better reduction ability and can reduce metal ions in one minute when compared with molecules with amine groups. More importantly, it is pH-responsive and consequently endows NPs prepared within it with pH-responsive properties. Here, HAuCl_4_ with different pH values was used to prepare Au NPs in order to investigate the influence of pH-sensitive acylhydrazone bonds on NP synthesis. The catalysis activities of metal (Au, Pt, Pd, Cu) NPs were characterized by p-nitrophenol (PNP) hydrogenation catalysis. Among them, Au prepared under under pH 4.0 and pH 6.7, Pt NPs, and Pd NPs show excellent catalysis activities for hydrogenation. Thus, an excellent catalyst system for hydrogenation catalysis based on multiarm hyperbranched polymer templated metal NPs was established.

## 2. Experimental

### 2.1. Materials

HAuCl_4_·3H_2_O (99.99%), H_2_PtCl_6_·6H_2_O (Pt 37.5%), K_2_PdCl_4_ (99.99%), CuSO_4_ (99.00%), and *p*-nitrophenol (PNP) were bought from Alfa Aesar (Lancashire, UK). HPAMAM-*g*-MPEG was from our lab. The ultrapure water with 18.2 MΩ·cm was used in all experiments.

### 2.2. Synthesis of Dynamic Hyperbranched Polymers with pH-Sensitive Acylhydrazone Bonds

#### 2.2.1. Synthesis of Hyperbranched Poly(Amidoamine) (HPAMAM)

Hyperbranched poly(amidoamine) (HPAMAM) can be synthesized from commercially available AB and C*n* types of monomers by one-pot polymerization via the coupled-monomer method [32]. 9.03 g (0.15 mol) ethylenediamine (EDA) in 28 mL methanol were put into a one-neck flask in an ice-salt bath, and then 25.83 g (0.3 mol) methyl acrylate (MA) was dropwise added to the flask under stirring. The mixture was stirred at room temperature for one week and then the flask was fixed onto a rotary evaporator to remove the methanol under the vacuum. After reacting for 1 h at 60 °C, 2 h at 100 °C, 2 h at 120 °C, and 2 h at 140 °C on the rotary evaporator in vacuum, a slightly yellow dope was obtained [32].

^1^H NMR (400 MHz, DMSO-d_6_, 298 K) δ: 2.10–2.30 (NH_2_), 2.30–2.90 (COCH_2_, NHCH_2_, NH_2_CH_2_), 2.91–3.30 (NCH_2_), 3.31–3.50 (NCH_2_), 3.51–3.62 (OCH_3_), 7.52, 7.97 (CONH).

#### 2.2.2. Synthesis of Acylhydrazine Terminated HPAMAM (HPAMAM-NHNH_2_)

0.5 g HPAMAM were dissolved in 40 mL anhydrous ethanol and then 20 mL 85% hydrazine hydrate was added. The mixture was stirred and refluxed at 125 °C for 3 h. After rotatory evaporating at 70 °C for 1 h and further drying in vacuum at 70 °C for 48 h, HPAMAM with acylhydrazine terminals (HPAMAM-NHNH_2_) was gained.

^1^H NMR (400 MHz, DMSO-d_6_, 298 K) δ: 1.83–2.30 (NH_2_), 2.30–2.80 (COCH_2_, NHCH_2_, NH_2_CH_2_), 2.86–3.24 (NCH_2_), 3.27–3.50 (NCH_2_), 3.62–4.40 (CONHNH_2_), 7.56, 8.13 (CONHCH_2_), 9.09 (CONHNH_2_).

#### 2.2.3. Synthesis of Multiarm Hyperbranched Polymers with pH-sensitive Acylhydrazone Bonds (HPAMAM-g-MPEG)

0.30 g HPAMAM-NHNH_2_ and 1.83 g *para*-substituted poly(ethylene glycol) monomethyl ether benzaldehyde (MPEG-C_6_H_4_CHO) were dissolved in anhydrous ethanol (50 mL). The ethanol solution was refluxed under N_2_ atmosphere for 24 h. After rotatory evaporating, the product was redispersed in dichloromethane (10 mL) and then drops were added to ice ether (200 mL). The resulting precipitate was dried under vacuum to obtain HPAMAM-g-MPEG.

^1^H NMR (400 MHz, DMSO-d_6_, 298 K) δ: 2.08–2.27 (NH_2_), 2.30–2.90 (COCH_2_, NHCH_2_, NH_2_CH_2_), 2.99–3.16 (NCH_2_), 3.23 (OCH_3_), 3.26–3.40 (NCH_2_), 3.44–4.22 (OCH_2_), 7.56, 8.13 (CONHCH_2_), 6.86–7.37 (Ar), 7.56–8.08 (CONHCH_2_), 8.63 (ArCH=N), 11.10–11.40 (CONHN=C) [38].

### 2.3. NP Synthesis within Multiarm Hyperbranched Polymer HPAMAM-g-MPEG

Typically, 7.5 mg HPAMAM-*g*-MPEG was dissolved in 3 mL ultrapure water and then 0.5 mL HAuCl_4_ (1.5 μmol) aqueous solution (pH value was adjusted to 2.9, 4.0 and 6.7 by 1 M NaOH for different samples, respectively) was added. After stirring for several minutes, claret-red Au NPs were obtained and abbreviated as Au/HPAMAM-*g*-MPEG. A similar procedure was done for Pt, Pd, and Cu NP synthesis except that the stirring time was all set as 48 h and HAuCl_4_ was replaced by equal amount of H_2_PtCl_6_, K_2_PdCl_4_ and CuSO_4_, respectively. For H_2_PtCl_6_ aqueous solution, the pH value was also adjusted to 6.7 by 1 M NaOH. UV-Vis spectra of Au, Cu, Pd, Pt nanoparticles synthesized within HPAMAM-g-MPEG can be seen in Figure S1.

### 2.4. PNP Hydrogenation Catalysis

The UV–Vis spectrophotometer was blanked with 2 mL of ultrapure water in a quartz cuvette and then 0.75 mL of 0.2 M NaBH_4_ and 0.25 mL of 600 μM PNP (pH = 12) aqueous solution were then added to the reaction cuvette. 150 μL of Au/HPAMAM-*g*-MPEG solution (pH = 11.0) was rapidly added into the reaction cuvette. Data were collected at a frequency of one per 1 second. The absorbance at 400 and 600 nm (for background correction) were used to calculate the kinetic rate constant by fitting to first-order integrated rate equations.

### 2.5. Measurements

Transmission electron microscopy (TEM) and elemental characterization were carried out on a JEOL 2010 microscope (JEOL, Tokyo, Japan) with an energy-dispersive X-ray spectrometer (JEOL, Tokyo, Japan) (EDS) at an accelerating voltage of 200 kV. UV–Vis spectra were recorded on a Varian Carry-50 UV–Vis spectrometer (JEOL, Tokyo, Japan).

## 3. Results and Discussion

**Scheme 1:**
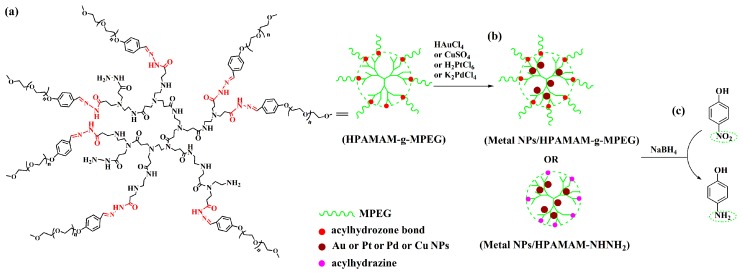
Illustration for the synthesis of Au, Pt, Pd, and Cu nanoparticles (NPs) within dynamic hyperbranched polymers HPAMAM-*g*-MPEG (**a**,**b**) and PNP hydrogenation via sodium borohydride in the presence of the metal catalyst (**c**).

**Figure 1:**
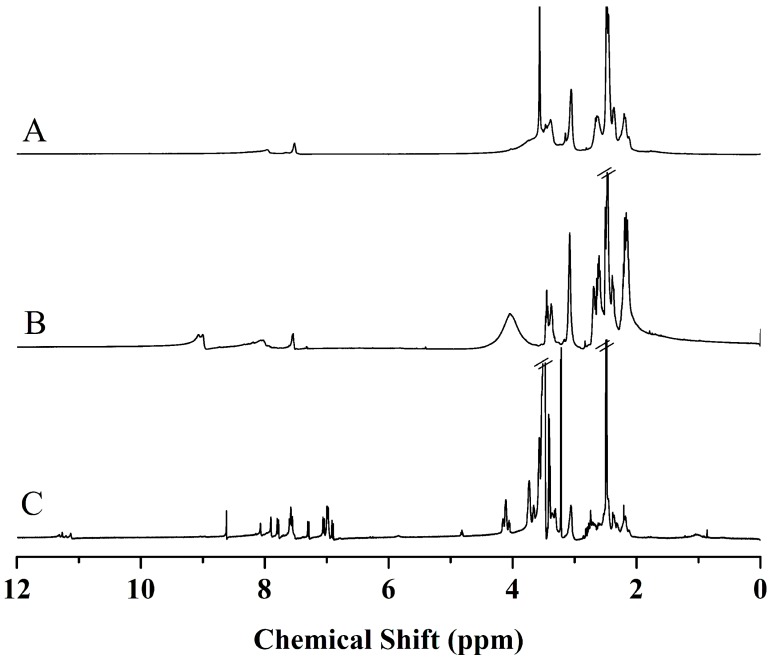
^1^H NMR spectra of (**A**) HPAMAM; (**B**) HPAMAM-NHNH_2_; and, (**C**) HPAMAM-*g*-MPEG (400 MHz, in DMSO-d6, 298 K).

Scheme 1a shows the chemical structure of HPAMAM-*g*-MPEG multiarm hyperbranched polymer used. HPAMAM-*g*-MPEG was constructed by combining acylhydrazine-modified hyperbranched poly(amidoamine) (HPAMAM-NHNH_2_) and para-substituted poly(ethylene glycol) monomethyl ether benzaldehyde (MPEG-C_6_H_4_CHO) by acylhydrazone bonds. The ^1^H NMR spectra of HPAMAM, HPAMAM-NHNH_2_ and HPAMAM-*g*-MPEG are shown in Figure 1. The signal at 3.58 ppm in Figure 1A corresponds to the methoxy terminals of HPAMAM, while it disappears in Figure 1B. This is because the methoxy terminals have been transformed into acylhydrazine after hydrazinolysis. After reacting with benzaldehyde-terminated MPEG, new proton signals at 8.63 and 11.10–11.40 ppm indicated the formation of acylhydrazone bonds, as shown in Figure 1C. HPAMAM-*g*-MPEG, a multiarm hyperbranched polymer with pH-sensitive acylhydrazone bonds, has great potential in synthesizing pH-responsive NPs, nanodots, etc. Here, HPAMAM-*g*-MPEG was used to prepare Au, Pt, Pd, and Cu NPs, as shown in Scheme 1b. The reversible performance of HPAMAM-g-MPEG can be seen in Figure S2. HPAMAM-*g*-MPEG firstly sequestered HAuCl_4_ (or H_2_PtCl_6_, or K_2_PdCl_4_ or CuSO_4_), and then reduced the metal ions into corresponding metal NPs by acylhydrazone bonds and amines, as shown in Scheme 1a,b.

**Figure 2:**
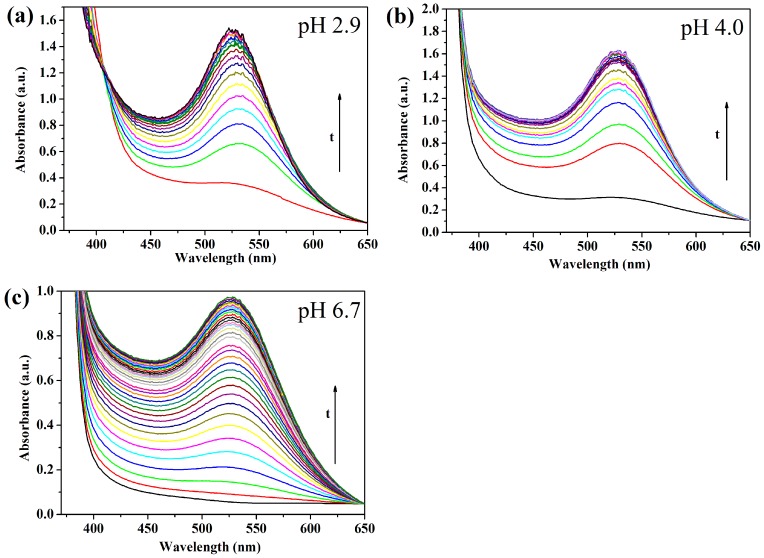
UV–Vis spectra of Au/HPAMAM-g-MPEG nanocomposites synthesized under different pH of HAuCl_4_ ((**a**) pH 2.9; (**b**) pH 4.0; and, (**c**) pH 6.7)).

Take HAuCl_4_ as an example, HPAMAM-*g*-MPEG can reduce all the HAuCl_4_ into Au NPs in two minutes, which was much quicker than other hyperbranched polymers with amines such as HPAMAM. We used HPAMAM-*g*-MPEG to reduce HAuCl_4_ with different values (pH 2.9, pH 4.0 and pH 6.7). Under pH 2.9 and pH 4.0, HPAMAM-*g*-MPEG can be cleaved into MPEG-CHO and HPAMAM-NHNH_2_, and metal NP/HPAMAM-NHNH_2_ would be formed, as shown in Scheme 1b. UV–Vis spectra were used to monitor the reduction rate. For the samples using HAuCl_4_ with pH 2.9 or pH 4.0 or pH 6.7, data were collected every 6 s, 12 s, and 3 min, respectively. The reduce time were 1.6, 3.4, and 92 min, respectively, as seen in Figure 2. As we know that acylhydrazone bonds in HPAMAM-*g*-MPEG can be cleaved into acylhydrazine and aldehyde groups under acid conditions (pH ≤ 5), while acylhydrazine have extraordinary strong reducing capacity, consequently, HAuCl_4_ aqueous solution with pH value lower than five can be reduced more quickly than that with high pH value.

**Figure 3:**
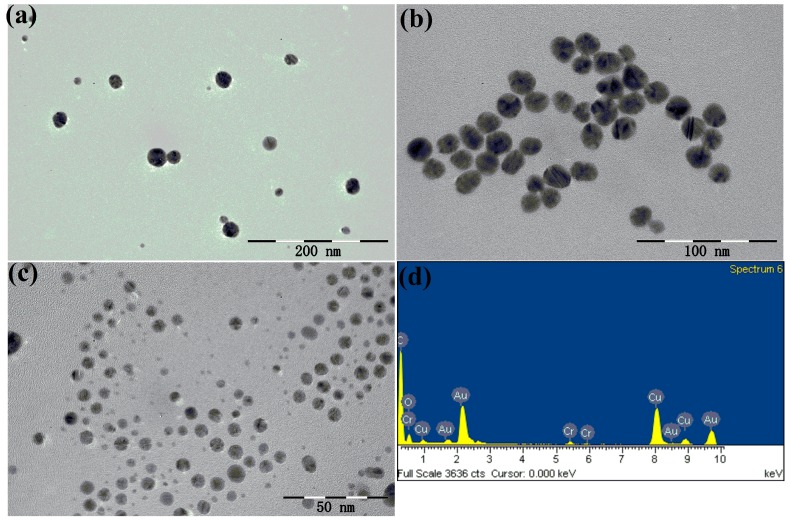
Transmission electron microscopy (TEM) images of Au NPs synthesized under different pH values of HAuCl_4_ ((**a**) pH 2.9; (**b**) pH 4.0; (**c**) pH 6.7) and energy-dispersive X-ray spectrometer (EDS) of Au NPs synthesized under pH 6.7 of HAuCl_4_ (**d**).

The morphology of Au NPs synthesized was characterized by TEM, as shown in Figure 3. The sizes of Au NPs synthesized under different pH of HAuCl_4_ (pH 2.9, pH 4.0, and pH 6.7) are 17.8 ± 6.6, 15.7 ± 2.0, and 5.3 ± 2.0 nm, respectively. Au NPs synthesized under pH 2.9 have a large size and wide size-distribution. This is because acylhydrazone bonds of HPAMAM-*g*-MPEG change into acylhydrazine groups, which could reduce HAuCl_4_ immediately and the resulting NPs cannot be well stabilized anymore. Au NPs synthesized under pH 4.0 have a low size-distribution when compared with that under pH 2.9 due to the lower cleavage rate of acylhydrazone bonds as compared to that under pH 2.9. Whereas, Au NPs synthesized under pH 6.7 have a rather smaller size when compared with that synthesized under other pH conditions. Under pH 6.7, acylhydrazone bonds remain stable and could reduce HAuCl_4_ gently. HPAMAM-*g*-MPEG not only acts as stabilizer but also as a reducing agent, resulting a small size and narrow size-distribution of Au NPs.

**Figure 4:**
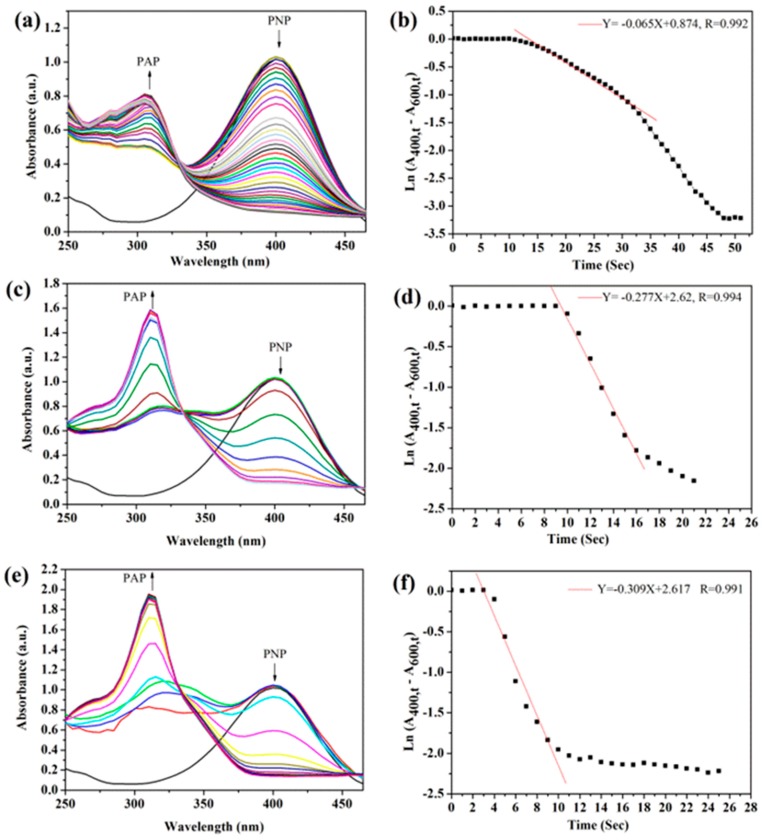
Reduction of p-nitrophenol (PNP) catalyzed by various Au NPs. (**a**), (**c**) and (**e**) show the UV–Vis spectra with arrows indicating the decreasing absorbance at 400 nm (peak of PNP) and the increasing absorbance at 310 nm (peak of PAP). (**b**), (**d**) and (**f**) show the plots of the natural log of corrected absorbance at 400 nm against time. (**a**,**b**) correspond to Au NPs synthesized at pH 2.9. (**c**,**d**) correspond to Au NPs synthesized at pH 4.0. (**e**,**f**) correspond to Au NPs synthesized at pH 6.7.

Metal NPs have significant potential as catalysts due to their high surface area and their size and shape-dependent catalytic activities. Here, the catalytic activities of Au NPs with various sizes were evaluated for PNP hydrogenation catalysis, as shown in Scheme 1c. The PNP, with a yellow color under alkaline conditions, is often reduced slowly into colorless *p*-aminophenol (PAP) by NaBH_4_. However, the reduction can be accelerated greatly in the presence of catalyst. The UV–Vis spectra were monitored every second, as shown in Figure 4a. The absorbance at 400 nm (peak of PNP) decreased, accompanied with an increase of 310 nm (peak of PAP). The PNP hydrogenation catalysis can also be evidenced by the color change from yellow to colorless. The kinetic rate constants were analyzed according to the first-order rate law [55,56,57,58,59,60]. The absorbance at 400 nm was subtracted from absorbance at 600 nm to correct for background absorption. By plotting the natural log of the corrected absorbance at 400 nm against time, and fitting the steepest part of the curve into a line, the slope, which is considered as the rate constant, can be obtained. The rate constants *k*_app_ for Au NPs synthesized under different pH values (pH 2.9, pH 4.0, pH 6.7) of HAuCl_4_ were 0.065, 0.277 and 0.309 s^−1^, respectively. The trend is consistent with the surface area of Au NPs. Au NPs prepared under pH 6.7 had the smallest diameter accompanying the largest surface area when compared with Au NPs prepared under other pH conditions, resulting in the highest rate constants *k*_app_ (s^−1^). When the rate constants were normalized to the surface area per unit volume, the rate constants *k_1_* were 0.92, 3.48, and 1.31 L·s^−1^·m^−2^, respectively. For the calculation of the hydrogenation rate constants *k*_app_ and k_1_ can be seen in Figure S3. For the volume, all of the catalysis experiments had the same volume. This means that the rate constants *k*_1_ is inversely proportional to the surface area of NPs, resulting in the biggest rate constants *k*_1_ for Au NPs prepared under pH 4 as compared to other Au NPs. Different from the G4 or G6 PAMAM dendrimers that have steric crowding problems at the periphery, HPAMAM-*g*-MPEG has a small HPAMAM core and several MPEG arms, so small molecules such as PNP can penetrate the MPEG shell and access the surfaces of small NPs more easily. That is why metal NPs synthesized within HPAMAM-*g*-MPEG have high rate constants. Detail information on the size of HPAMAM and HPAMAM-g-MPEG measured by DLS can be seen in Figure S4.

**Figure 5:**
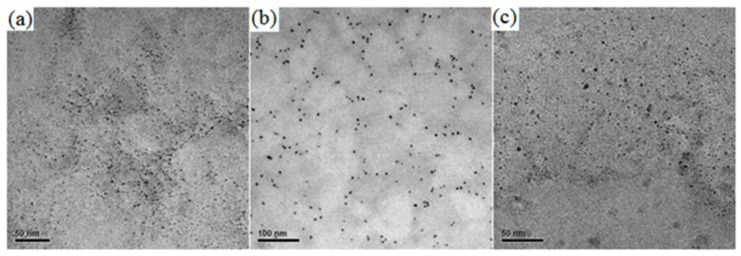
TEM images of Pt (**a**), Pd (**b**), and Cu (**c**) NPs synthesized within the multiarm hyperbranched polymer (HPAMAM-*g*-MPEG).

As reported in many papers [55,56,57,58,59,60], Pt and Pd NPs have also been shown to be very efficient catalysts for hydrogenation and Heck reactions. Consequently, dynamic HPAMAM-*g*-MPEG was also used as reductant and nanoreactor to prepare Pt, Pd, and Cu NPs. The TEM images of Pt, Pd, and Cu NPs are shown in Figure 5. The average diameter of Pt, Pd, and Cu NPs are 3.7, 5.9, and 4.2 nm, respectively.

**Figure 6:**
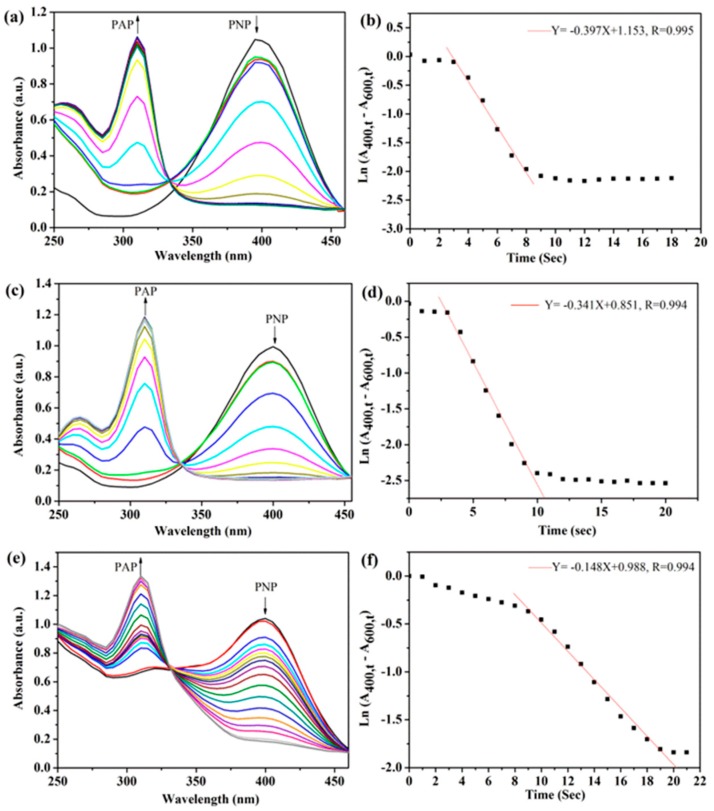
Reduction of PNP catalyzed by Pt (**a**,**b**), Pd (**c**,**d**), and Cu (**e**,**f**) NPs. (**a**,**c**,**e**) show the UV–Vis spectra with arrows indicating the decreasing absorbance at 400 nm (peak of PNP) and the increasing absorbance at 310 nm (peak of PAP). (**b**,**d**,**f**) show the plots of the natural log of corrected absorbance at 400 nm against time.

The catalytic activities of Pt, Pd, and Cu NPs synthesized within HPAMAM-*g*-MPEG were also evaluated by PNP hydrogenation catalysis, as shown in Figure 6. The rate constants *k*_app_ for Pt, Pd, and Cu NPs were 0.397, 0.341, 0.148 s^−1^, respectively. After being normalized to the surface area per unit volume, the rate constants *k*_1_ were 1.86, 1.32, and 0.72 L·s^−1^·m^−2^, respectively. From the rate constant, we can see that Pt and Pd NPs have better catalysis activities as compared with that of Cu NPs. As reported in many papers [55,56,57,58,59,60], noble Pt and Pd NPs are commonly used as catalysts for catalytic hydrogenation, Heck reaction and Suzuki-coupling reaction, and always have good catalysis activities. For Cu NPs, their undesirable catalysis behavior can be attributed to their ease oxidation property. Information on catalysis rate constants of NPs has been summarized in Table S1.

## 4. Conclusions

In this paper, size-controlled Au NPs were prepared within multiarm hyperbranched polymer HPAMAM-*g*-MPEG under different pH of HAuCl_4_. HPAMAM-*g*-MPEG, not only was used as reductant to reduce metal ions, but also can be used as nanoreactors and stabilizers to prepare metal NPs. Systematic investigation of the effect of NP size and the pH value of HAuCl_4_ on the catalytic activity of the resulting Au NPs was performed. The rate constants *k*_1_ for Au NPs prepared under 4.0 of HAuCl_4_ could reach as high as 3.48 L·s^−1^·m^−2^ for the reduction of PNP. We suppose that this is because the HPAMAM-NHNH_2_ generated from the cleavage of HPAMAM-*g*-MPEG under acid conditions has high reducing compatibility and low steric crowding problems at the periphery. Pt, Pd, and Cu NPs were also synthesized within multiarm hyperbranched HPAMAM-*g*-MPEG, and also proved to be highly active catalysts for the hydrogenation reaction. For Pt NPs, the rate constants *k_1_* was as high as 1.86 L·s^−1^·m^−2^ for the reduction of PNP. The results presented here demonstrate the utility of multiarm hyperbranched polymer as nanoreactor for preparing novel metal NPs and their excellent catalytic properties. The metal NP/HPAMAM-*g*-MPEG nanocomposites would be very promising catalysts in many conditions.

## Supplementary Materials

The following are available online at www.mdpi.com/2073-4360/9/9/459/s1.
