# Supplementary material for: Catalyst System for Hydrogenation Catalysis Based on Multiarm Hyperbranched Polymer Templated Metal (Au, Pt, Pd, Cu) Nanoparticles
Source: Polymers (Basel). 2017 Sep 19;9(9):459. doi: 10.3390/polym9090459 (PMC6418630; doi:10.3390/polym9090459)
Supplement: Supplementary file 1 [file polymers-09-00459-s001.pdf]

Supporting Information

Catalyst System for Hydrogenation Catalysis based on Multiarm Hyperbranched Polymer Templated Metal (Au, Pt, Pd, Cu) Nanoparticles

Yunfeng shi, Lixin Liu, Fengyue Zhang, Mengyuan Niu, Yanzhu Zhao, Yifan Fan, Yanping Liang, Mei Liu, Zhenzhu Zhang and Junjie Wang

Contents Pages

Part I: Test conditions and result analysis..S2-4
SII-1. UV-Vis spectra..S2
SII-2. Reversible performance test on HPAMAM-g-MPEG...S2-3
SII-3. Calculation method for the hydrogenation rate constants k_1...S4
SII-4. Table S1. Information on molecular weight, PDI and size of polymer, nanoparticle size synthesized under various pH and catalysis rate constants of NPs..S5
SII-5. Dynamic light scattering (DLS) measurements..S6

Part I: Test conditions and result analysis

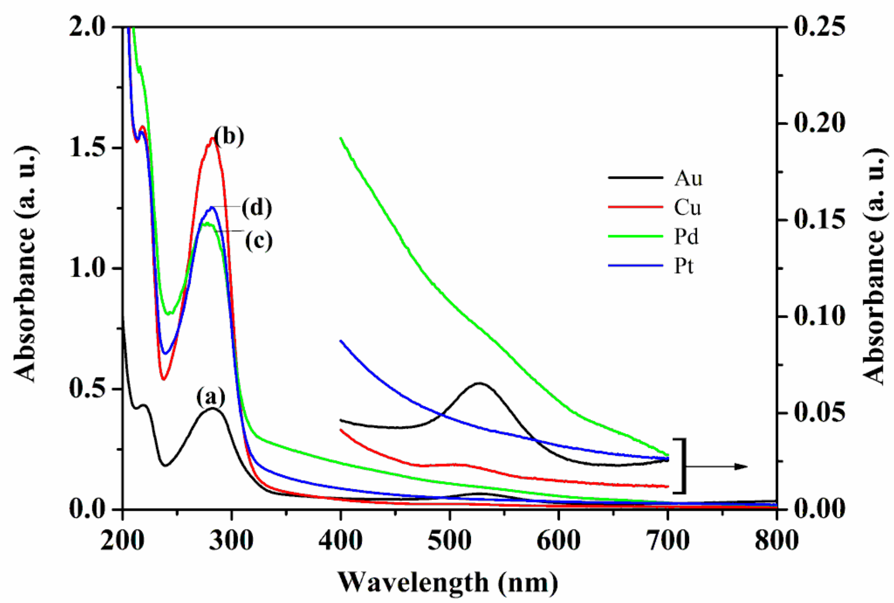

Figure S1. UV-Vis spectra of Au (a), Cu (b), Pd (c), Pt (d) nanoparticles synthesized within HPAMAM-g-MPEG.

The absorption peak of HPAMAM-g-MPEG locates at 219 and 283 nm. Au nanoparticles synthesized under pH 6.7 and Cu nanoparticles have absorption peaks at 527 nm, 509 nm, respectively.

The reversible performance test on materials was tested. Figure S2 shows the hydrolysis process of HPAMAM-g-MPEG in an acetate buffer D_2O solution (pH 3.0) through the ^1H NMR technique. It can be found that the proton signals of the benzaldehyde terminal at 9.65 ppm occurred after 0.5 h. The signals at 9.65 ppm show no difference after 3 h and 24 h, indicating that acylhydrazone bonds in HPAMAM-g-MPEG had been fully cleaved in 0.5 h. After hydrolysis, the hydrolyzed HPAMAM-g-MPEG was heated under vacuum for 24 h and then measured by ^1HNMR (D_2O). From figure S2(d), we can see that the signals at 9.65 ppm diminish and new signals at 11.10-11.40 (CON*H*N=C) occur, indicating acylhydrazone bonds were formed again. From these results, we can see that HPAMAM-g-MPEG has reversible performance.

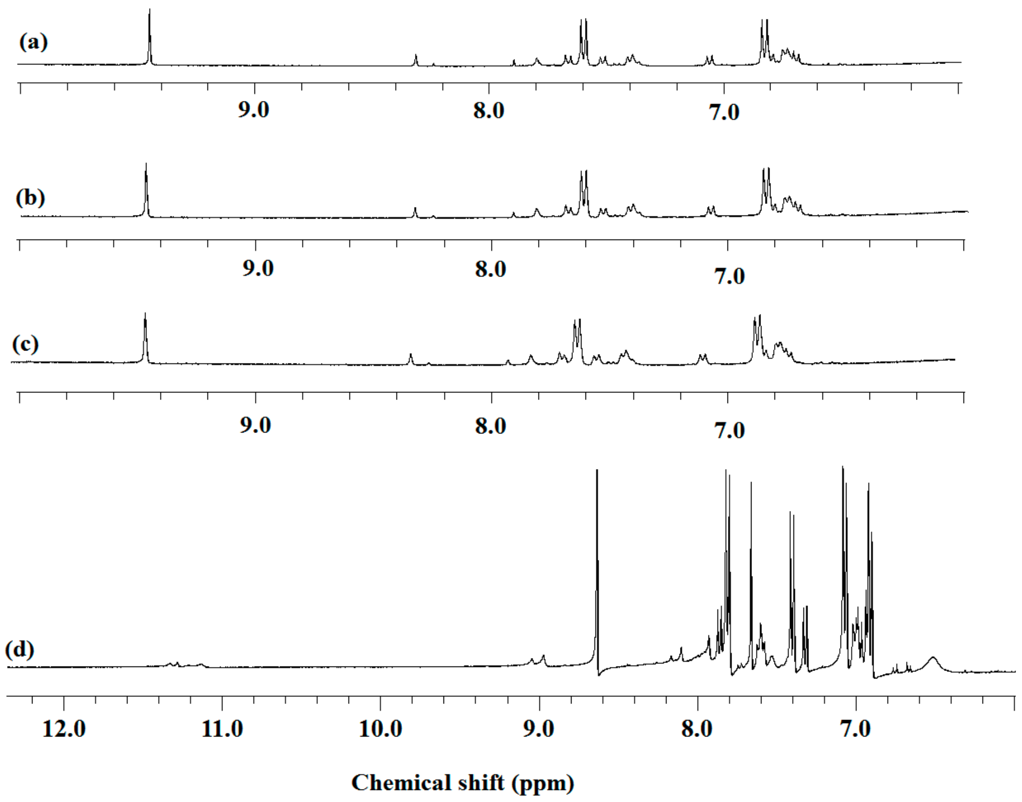

Figure S2. Hydrolysis of HPAMAM-g-MPEG in an acetate buffer D_2O solution at pH=3.0 (400MHz, D_2O, 298 K). (A) ^{1}HNMR spectra of HPAMAM-g-MPEG at different times: (a) 0.5 h, (b) 3 h and (c) 24 h. (d) ^{1}HNMR spectrum of hydrolyzed HPAMAM-g-MPEG after heating under vacuum for 24 h.

The calculation method for the hydrogenation rate constants k_1 can be seen as follows:

$$k_1 = \frac{k_{app}}{S/V}$$

k_{app}: rate constants (S^{-1})

S: surface area of metal nanoparticles used in the catalysis experiment (m^2)

V: the total volume of the catalysis experiment (L)

$$S = \frac{n * M}{4/3\ \pi\ r^3 * \rho} \times 4\ \pi\ r^2 \times 10^3$$

S: surface area of metal nanoparticles (m^2)

n: mol of metal atoms (mol)

M: atom weight of metal

r: radius of metal nanoparticles (nm)

ρ: density of metal nanoparticles (g/cm^3)

n * M: weight of metal atoms (g)

$4/3\ \pi\ r^3\ \rho$: weight of one metal nanoparticle (g)

$4\ \pi\ r^2$: surface area of one metal nanoparticle (nm^2)

Figure S3. Calculation of the hydrogenation rate constants k_1.

Table S1. Information on molecular weight, PDI and size of polymer, nanoparticle size synthesized under various pH and catalysis rate constants of NPs.

Sample	Mw (10^4)	PDI (Mw/Mn)	Size (nm)	catalysis rate constants (k_{app}, s^{-1})	catalysis rate constants (k_1, L s^{-1} m^{-2})
HPAMAM-g-MPEG	1.63	1.42	3.9 [a]	/	/
Au NPs synthesized under pH 2.9, pH 4.0, pH 6.7 of $HAuCl_4$	/	/	17.8±6.6	0.065	0.92
Au NPs synthesized under pH 4.0 of $HAuCl_4$	/	/	15.7±2.0	0.277	3.48
Au NPs synthesized under pH 6.7 of $HAuCl_4$	/	/	5.3±2.0	0.309	1.31
Pt NPs	/	/	3.7	0.397	1.86
Pd NPs	/	/	5.9	0.341	1.32
Cu NPs	/	/	4.2	0.148	0.72

[a] Measured by dynamic light scattering (DLS).

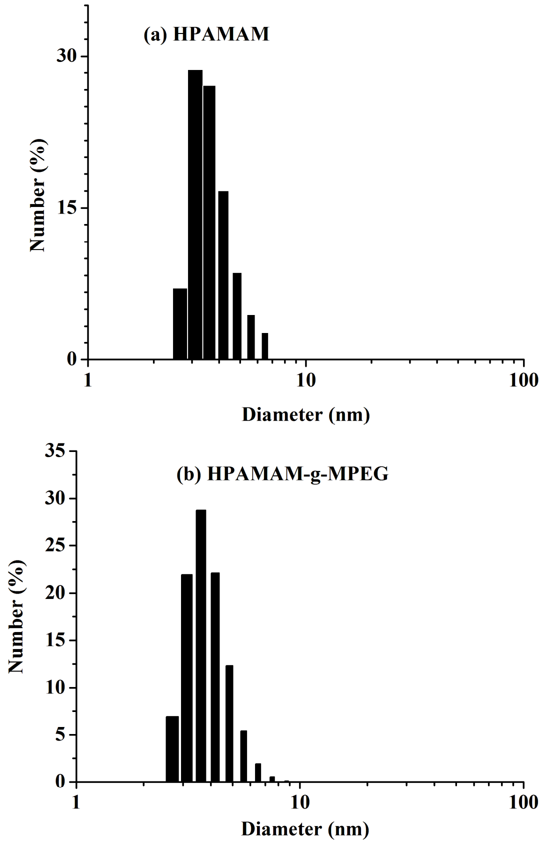

Figure S4. Dynamic light scattering (DLS) measurements of the hydrodynamic diameters of (a) HPAMAM and (b) HPAMAM-g-MPEG.

For HPAMAM, its weight-average molecular weight is 3700 g/mol and has a diameter of 3.1 nm measured by DLS. After grafting several MPEG, the weight-average molecular weight of HPAMAM-g-MPEG is 16300 g/mol and has a diameter of 3.9 nm measured by DLS.
